# Vulnerable leadership

**DOI:** 10.1080/17571472.2016.1163939

**Published:** 2016-05-02

**Authors:** Louise Younie

**Affiliations:** ^a^ Clinical Senior Lecturer, QMUL, London, UK

## Why this matters to me

Vulnerability is often considered a weakness and something to avoid especially within the competitive medical culture, yet my belief and experience is to the contrary. It seems to me those in leadership who can engage with their own vulnerability are potentially exercising great strength and courage as well as bringing relief and release for others looking up to them. Also in engagement with patients, being able to touch our own vulnerability allows us to draw closer to those forced into vulnerable situations through situations beyond their control.

## Key message

This paper challenges the notion of the powerful doctor and vulnerable patient, instead highlighting the gift of our shared vulnerability and humanity.

At a medical humanities educator workshop, a few years ago, we asked participants to bring an image or poem that they resonated with from the ‘Out of Our Heads’ website. ^1^ Images and words were shared with the group along with why they had been selected. Towards the end of our semi-circle was a senior clinician and an educator. His chosen image was one of a patient, sat in foetal position on a waiting room chair (http://www.outofourheads.net/oooh/handler.php?id=459). He had chosen this image because it reminded him of the patients’ vulnerability. He paused. It reminded him also of his own vulnerability.

During the coffee break a fifth-year medical student shared with me how his words had been the most useful utterance she had heard for months, having just completed her finals. I probed a little deeper. She told me of student stress and bravado. To hear someone further down the line able to talk about his own vulnerability was, to her, like a breath of fresh air.

I have pondered this anecdote for a number of years in the context of medical education and medical practice, and more recently in the light of my own dance with death through cancer. Why is it, I wonder, that we so rarely share our experiences of vulnerability either as students or qualified health professionals? I know from my own experiences that vulnerability and being a patient go hand in hand. As a patient there is little choice, but to embrace our fragile state when our mortal body has been assailed by some kind of pestilence. But for the healthy doctor who wields power in the lives of those struck by disease, vulnerability can be and often is held at arm’s length. Research suggests, however, that feelings of professional uncertainty do abound especially for students and young doctors in emotionally laden situations.[1] So it may be that we feel vulnerable but do not share or talk about it. A student writes about sitting in a breast cancer clinic and in one day hearing three people receive a diagnosis of breast cancer, the youngest being just a couple of years older than herself:

We were discussing how it is almost taboo to show emotion about things like this, not only on the wards but also when chatting afterwards to other students. There is an unwritten rule that you have to prove … that you can cope with hearing/having to give bad news and be absolutely fine with it. That’s the mark of a good doctor- leaving it all in the hospital.[2]

Anatole Broyard [3], a writer who died of prostate cancer disagrees. He invites the doctor to ‘let the sick man into his heart’, to share ‘the wonder, terror and exaltation of being on the edge of being’. That is where our shared humanity lies. When doctors distance themselves and batten down the hatches they may never get beyond their own shores of understanding, and their patients are left to voyage the seas unaccompanied and alone. Brene Brown [4] is a researcher and storyteller who has written a great deal in the field of vulnerability. She postulates that allowing ourselves to be seen and known, that is, making ourselves vulnerable, allows us to connect in meaningful ways with others. A leadership website explores the balance of strength and vulnerability and how shared vulnerability can lead to inclusiveness, teamwork and enhanced credibility.[5] Vulnerability in the context of leadership, says another site, ‘implies the courage to be yourself’, ‘replacing professional distance … with uncertainty, risk and emotional exposure.[6] These ideas could apply equally well to interactions with patients or peers.

My colleague at the educational workshop who shared the vulnerability that he can feel as a doctor flies in the face of the prevailing medical culture. His words worked to build community and connection. He shows his soft underbelly, strong and courageous enough to leave himself exposed and open to attack. Henri Nouwen [7], a Christian mystic, offers a perspective of vulnerability as strength and a gift.

When we honestly ask ourselves which person in our lives mean the most to us, we often find that it is those who, instead of giving advice, solutions, or cures, have chosen rather to share our pain and touch our wounds with a warm and tender hand. The friend who can be silent with us in a moment of despair or confusion, who can stay with us in an hour of grief and bereavement, who can tolerate not knowing, not curing, not healing and face with us the reality of our powerlessness, that is a friend who cares.

We could change the word ‘friend’ above, for ‘doctor’, and although we are often called upon to give advice, to lead and be ‘strong’, the strength of sharing vulnerable spaces may be one of the most generous things we can offer our peers, students or patients.

## Disclosure statement

No conflicts of interest.

## Footnotes

^1^
www.outofourheads.net. Creative reflective work is collated from medical students predominantly at the University of Bristol. There is also staff and patient contributions on this website.
